# Supplementary material for: Reduced Low–Pressure Membrane Fouling by Inline Coagulation Pretreatment for a Colored River Water
Source: Membranes (Basel). 2022 Oct 22;12(11):1028. doi: 10.3390/membranes12111028 (PMC9695621; doi:10.3390/membranes12111028)
Supplement: Supplementary file 1 [file membranes-12-01028-s001.zip › membranes-1984890-supplementary.pdf]

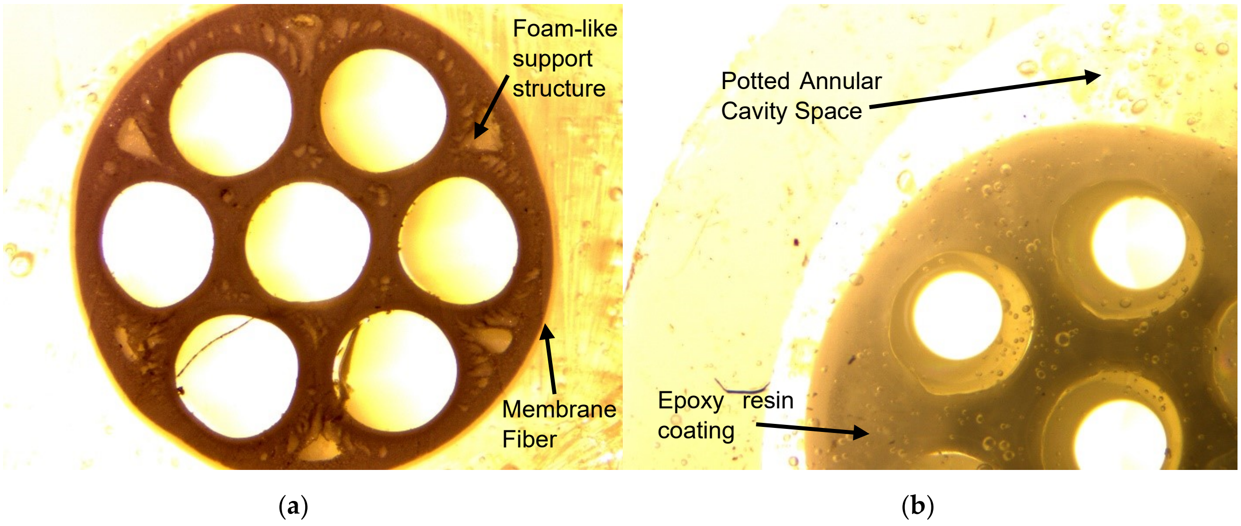

Figure S1. Potted tubular monolith fiber: **(a)** prior to sealing foam-like support structure; **(b)** after sealing support structure.

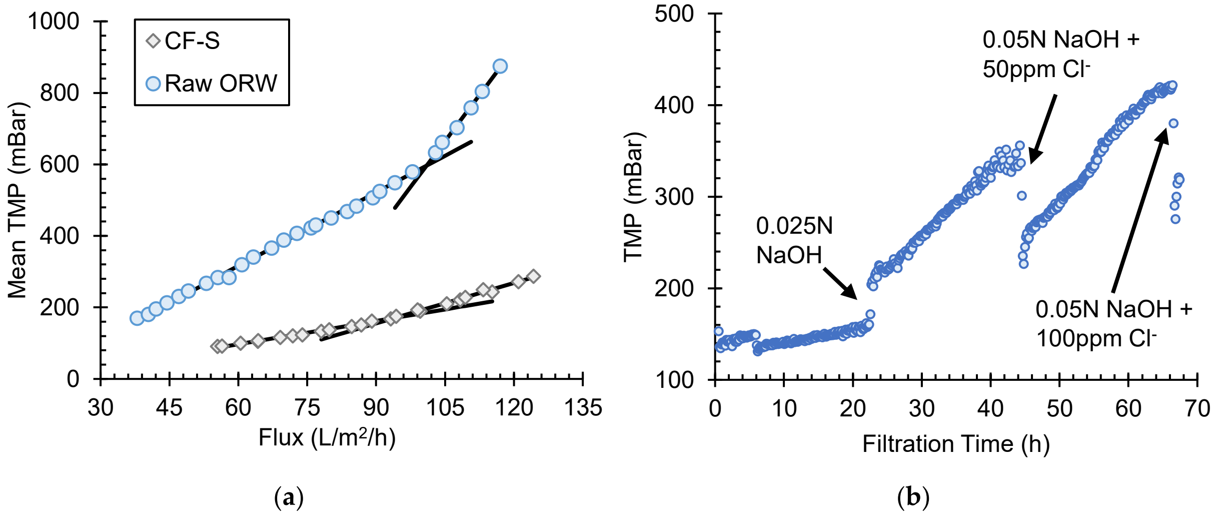

Figure S2. Preliminary testing: **(a)** critical flux determination; **(b)** CEB optimization.

$$FR_{hr} = \frac{TMP_{n-1,f} - TMP_{n,i}}{t_{n-1,f} - t_{n-1,i}}$$

$$FR_{hirr} = \frac{TMP_{n,i} - TMP_{n-1,i}}{t_{n-1,f} - t_{n-1,i}}$$

$$FR_{CEB} = \frac{TMP_k - TMP_{k-1}}{t_k - t_{k-1}}$$

$$FR_{CIP} = \frac{TMP_3 - TMP_0}{t_3}$$

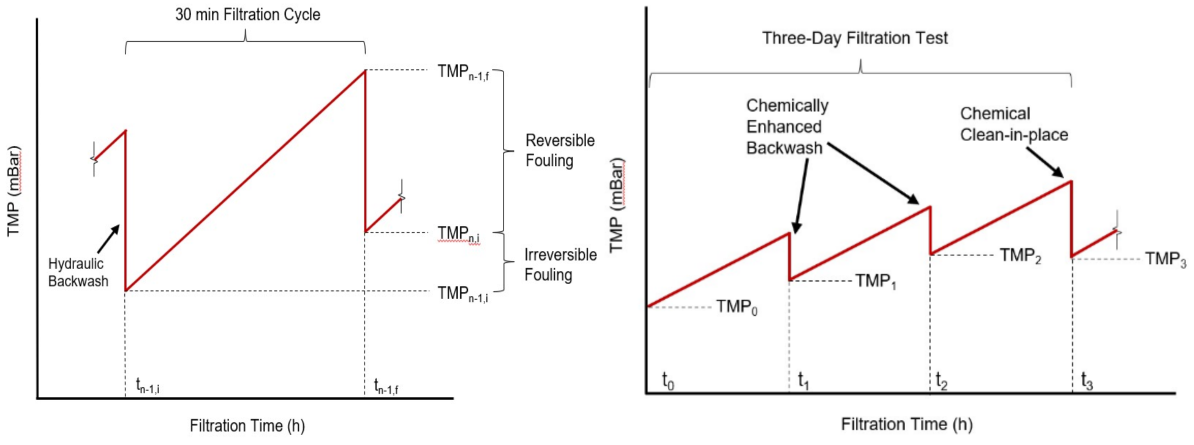

(**a**) (**b**)

Figure S3. Fouling rate computation: **(a)** individual filtration cycle reversible and irreversible fouling rate; **(b)** chemically irreversible fouling rates.

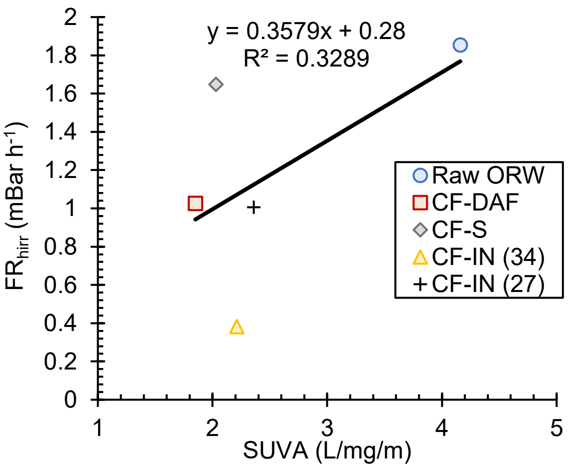

Figure S4. Relationship between FR_{irr} and SUVA.

Table S1. TMC membrane fouling rates.

Parameter	Raw ORW	CF-DAF	CF-S	CF-IN (34 mg L^{-1})		CF-IN (27 mg L^{-1})	
	Winter	Winter	Winter	Winter	Summer	Winter	Summer
FR_{hr} (mBar h^{-1})	35.1 ± 1.12	9.77 ± 0.50	8.50 ± 0.44	12.5 ± 1.0	11.7 ± 0.51	9.23 ± 0.53	16.8 ± 0.82
FR_{hirr} (mBar h^{-1})	1.85 ± 0.51	1.03 ± 0.38	1.65 ± 0.43	0.46 ± 0.27	0.28 ± 0.43	1.0 ± 0.59	0.25 ± 0.41
FR_{TOT} (mBar h^{-1})	37 ± 1.15	10.8 ± 0.61	10.2 ± 0.55	13 ± 1.02	11.9 ± 0.56	10.2 ± 0.71	17.1 ± 0.84
FR_{CEB} (mBar h^{-1})	0.474	0.615	0.481	0.014	0.256	0.516	0.132
FR_{CIP} (mBar h^{-1})	0.880	0.815	0.420	0.027	0.190	0.683	0.119
